# Supplementary material for: Diagnostic efficacy of the magnetic resonance T1w/T2w ratio for the middle cerebellar peduncle in multiple system atrophy and spinocerebellar ataxia: A preliminary study
Source: PLoS One. 2022 Apr 15;17(4):e0267024. doi: 10.1371/journal.pone.0267024 (PMC9012356; doi:10.1371/journal.pone.0267024)
Supplement: Attachment — Submitted filename: Response to Reviewers.docx [file pone.0267024.s002.docx]

04/11/2021

Prof. Emily Chenette

Editor-in-Chief

*PLOS ONE*

Dear Prof. Chenette:

We greatly appreciate your review of our manuscript and the helpful suggestions. We have now revised the manuscript according to the reviewers’ comments. Changes made in accordance with the reviewers’ comments are indicated by red text in the revised manuscript. In your email of September 20, you recommended including additional scans acquired with a protocol that is more similar to what is typically done with T1w/T2w ratio. However, scans with such protocol have not been taken in the patients with SCA3 or SCA6 in our department so far. Instead, we have added a control group to the current study and have shown that diagnostic accuracy in differentiating MSA from controls is comparable to a previous study using 3T MRI (Sugiyama A, et al. Eur Radiol 2021;31:4277-4284).

We hope that you will consider this revised version suitable for publication.

Sincerely,

Dr. Atsuhiko Sugiyama, MD, PhD
Department of Neurology, Graduate School of Medicine, Chiba University

Phone No: +81-43-226-2129

Fax No: +81-43-226-2160

Email Address: [asugiyama@chiba-u.jp](mailto:asugiyama@chiba-u.jp)

# Reply to Reviewer 1

We greatly appreciate your helpful comments and suggestions. Changes made in accordance with reviewers’ comments are indicated in red font in the revised manuscript.

*#1 Was there a correlation between MRI findings suggestive of MSA-C and the presence of a REM sleep behavior disorder?*

Reply: Unfortunately, there was no systematic evaluation of RBD using questionnaires or PSG in the patients with MSA included in this study. Moreover, some patients were not asked about their RBD since little attention had been paid to the relationship between RBD and MSA in the past. We have added a note to the Discussion section about this as a limitation, as follows.

Page 21, line 348-351

“Third, owing to the retrospective nature of this study, the relationship between the MCP sT1W/T2W ratio and rapid eye movement sleep behavior disorder or clinical scales for cerebellar ataxia, such as the International Cooperative Ataxia Rating Scale or Scale for the Assessment and Rating of Ataxia, could not be evaluated.”

*#2 Was there a correlation between MRI findings suggestive of MSA-C and the SARA ataxia scale?*

Reply: We agree with the reviewer’s point that assessing the correlation between MRI findings and cerebellar symptom rating scales, including SARA, is important. Unfortunately, no systematic assessment of cerebellar symptoms, including SARA, has been performed in the patients with MSA included in this study. We have added a note to the Discussion section about this as a limitation, as follows. We hope to evaluate the correlation between SARA and MRI findings in future prospective studies.

Page 21, line 348-351

“Third, owing to the retrospective nature of this study, the relationship between the MCP sT1W/T2W ratio and rapid eye movement sleep behavior disorder or clinical scales for cerebellar ataxia, such as the International Cooperative Ataxia Rating Scale or Scale for the Assessment and Rating of Ataxia, could not be evaluated.”

*#3 Without a doubt, a prospective study would be very suitable to analyze the results of this study in greater depth, as well as with a greater number of cases of MSA-C and the group of patients with SCAs 3 and 6.*

Reply: We agree with the reviewer’s comment and have revised and added sentences in the Discussion section, as follows.

Page 20, line 336

“This study has several limitations.”

Page 21, line 352-355

“Fourth, the sample size of the SCA3 group was small (n = 8), and future prospective studies with larger sample sizes that evaluate clinical scales for cerebellar ataxia are needed to validate the findings of this study and clarify the relationship between clinical severity of cerebellar ataxia and the MCP sT1W/T2W ratio.”

# Reply to Reviewer 2

We greatly appreciate your helpful comments and suggestions. Changes made in accordance with reviewers’ comments are indicated in red font in the revised manuscript.

*#1 One of the major concerns of the paper is the data used. The results might be biased mostly by noise. Data used in this work is 2D and image resolutions are not the same for T1w and T2w images and data was collected with 1.5 T MRI which hinherently has a low signal-to-noise ratio.*

Reply: We agree with the reviewer’s concerns about bias in the data due to the MRI sequences and magnetic field strength of the MRI scanner used in this study. We have added sentences in the Discussion section as a limitation on this point, as follows.

Page 20, line 336 to Page 21 line 346

“The first is that 2D T2W images were co-registered with 3D T1W images, which do not have the same image resolution, and a 1.5-T MRI scanner with an inherently low signal-to-noise ratio was used; therefore, the results of the MCP sT1W/T2W ratio may be biased by noise. However, the MCP sT1W/T2W ratio values in patients with MSA-C correlated well with the extent of MCP hyperintensities and MCP volume, suggesting that the MCP sT1W/T2W ratio values measured in this study well reflected the degeneration of the MCP in MSA-C. Moreover, the diagnostic accuracy of the MCP sT1W/T2W ratio in discriminating MSA-C from controls in this study was comparable to that observed in a study using a 3-T MRI scanner [16]. Further studies employing 3D T1WI and T2WI sequences using a 3-T MRI scanner, which might allow better quantification of the sT1W/T2W ratio, are needed to confirm the results of this study.”

*#2 The authors haven’t clearly mentioned what they are measuring with T1w/T2w and how sensitive the method is to tissue microstructure especially the myelin. T1w/T2w method is considered to be sensitive to myelin but depends on how T1w and T2w images are acquired as well as the image processing pipeline. For better quantification of T1w/T2w, 3D T1w and 3D T2w SPACE sequences are recommended. A recent work by Uddin et al (NMR in Biomedicine 2018) reported that TSE based T1w/T2w ratio is not a good measure to quantify myelin.*

Reply: We want to thank the reviewer for this insightful comment. According to this reviewer’s comment, we have added sentences in the Discussion section, as follows.

Page 19, line 294-308

“Decreased MCP sT1W/T2W ratio values in patients with MSA-C might reflect demyelination and gliosis in the MCP due to MSA-C-related degeneration. Pathologically, the loss of axons, myelin, and reactive gliosis was observed in the MCP of patients with MSA-C [28]. The sT1W/T2W ratio is sensitive to myelin and decreases in demyelinating lesions [29, 30]. In contrast, inconsistent correlations with histology [31], myelin water imaging [12, 32], simultaneous tissue relaxometry, and magnetization transfer saturation index [33] suggest that the sT1W/T2W ratio is sensitive to not only myelin content but also other microstructural factors. Moreover, gliosis is presumed to affect the sT1W/T2W ratio in a downward direction, mainly by prolonged T1 relaxation time [34]. Therefore, although the sT1W/T2W ratio might not be specific for myelin content, it might be more sensitive than evaluating MCP hyperintensities on T2WI to detect degenerative processes in the MCP, such as demyelination and gliosis associated with MSA-C. In this study, the MCP sT1W/T2W ratio had better diagnostic accuracy in discriminating MSA-C from SCA3 than MCP volume. This result is consistent with those of previous pathological studies, showing that gliosis and demyelination precede atrophy in MSA [35, 36].”

In line with this revision, we have added references.

28. Itoh K, Kasai T, Tsuji Y, Saito K, Mizuta I, Harada Y, et al. Definite familial multiple system atrophy with unknown genetics. Neuropathology. 2014;34:309-13.

29. Glasser MF, Goyal MS, Preuss TM, Raichle ME, Van Essen DC. Trends and properties of human cerebral cortex: correlations with cortical myelin content. NeuroImage. 2014;93:165-75.

30. Nakamura K, Chen JT, Ontaneda D, Fox RJ, Trapp BD. T1-/T2-weighted ratio differs in demyelinated cortex in multiple sclerosis. Ann Neurol. 2017;82:635-9.

31. Righart R, Biberacher V, Jonkman LE, Klaver R, Schmidt P, Buck D, et al. Cortical pathology in multiple sclerosis detected by the T1/T2-weighted ratio from routine magnetic resonance imaging. Ann Neurol 2017;82:519-29.

32. Uddin MN, Figley TD, Marrie RA, Figley CR; For the CCOMS Study Group. Can T1w/T2w ratio be used as a myelin-specific measure in subcortical structures? Comparisons between FSE-based T1w/T2w ratios, GRASE-based T1w/T2w ratios and multi-echo GRASE-based myelin water fractions. NMR Biomed. 2018;31:e3868.

33. Hagiwara A, Hori M, Kamagata K, Warntjes M, Matsuyoshi D, Nakazawa M, et al. Myelin measurement: comparison between simultaneous tissue relaxometry, magnetization transfer saturation index, and T1w/T2w ratio methods. Sci Rep. 2018;8:10554.

34. Barnes D, McDonald WI, Landon DN, Johnson G. The characterization of experimental gliosis by quantitative nuclear magnetic resonance imaging. Brain. 1988;111:83-94.

35. Jellinger KA, Seppi K, Wenning GK. Grading of neuropathology in multiple system atrophy: proposal for a novel scale. Mov Disord. 2005;20:29-36.

36. Kon T, Mori F, Tanji K, Miki Y, Wakabayashi K. An autopsy case of preclinical multiple system atrophy (MSA-C). Neuropathology. 2013;33:667-72.

*#3 The authors mentioned about T2 hyperintensities in MCP. I was wondering if they have any scores for WM hyperintensities or disease severity scores that can be correlated with T1w/T2w.*

Reply: We agree with the reviewer’s point that assessing the correlation between MCP sT1W/T2W ratio values and scores for MCP hyperintensities or disease severity scores, such as SARA, is important. We have assessed the MCP hyperintensities semiquantitatively and evaluated the correlation between the MCP sT1W/T2W ratio and MCP hyperintensities. In line with this revision, we have added sentences in the Methods and Results sections. Unfortunately, no systematic assessment of cerebellar symptoms, including SARA, has been performed in the patients with MSA included in this study. We have added a note to the Discussion section about this as a limitation.

Page 7, line 159-161

“A board-certified neuroradiologist (H. Y.), who was blinded to the clinical data, semiquantitatively evaluated the extent of MCP hyperintensities in the patients with MSA by applying the following scores: 0 = absent, 1 = mild, 2 = moderate, and 3 = severe.”

Page 9, line 208-210

“Spearman correlation analysis was performed to evaluate the relationship between the extent of MCP hyperintensities assessed semiquantitatively and the MCP sT1W/T2W ratio values in patients with MSA-C.”

Page 12, line 256-258

“In patients with MSA-C, a significant inverse correlation between the extent of MCP hyperintensities and MCP sT1W/T2W ratio was observed (*r* = −0.745; *p* < 0.001).”

Page 21, line 348-351

“Third, owing to the retrospective nature of this study, the relationship between the MCP sT1W/T2W ratio and rapid eye movement sleep behavior disorder or clinical scales for cerebellar ataxia, such as the International Cooperative Ataxia Rating Scale or Scale for the Assessment and Rating of Ataxia, could not be evaluated.”

*#4 MRI acquisition: data were acquired with a 1.5 T scanner. Please add information about the RF coil (i.e, 32-channel head coil), number of averages, number of slices.*

Reply: According to the reviewer’s comments, we have revised sentences in the Discussion section, as follows.

Page 5, line 114-120

“All MR images were acquired using a 1.5-T MRI system (Genesis Signa; GE Healthcare) with a 12-channel head coil. The MRI parameters for T1WI were as follows: 3D-IR-SPGR; sagittal plane; TR, 8 ms; TE, 3 ms; flip angle, 15°; FOV, 220 × 220 mm; matrix, 256 × 256; voxel size, 0.86 × 0.86 × 1.5 mm; number of slices, 248; and number of averages, 1. Meanwhile, those for T2WI were as follows: 2D-TSE; TR, 4000 ms; TE, 106 ms; FOV, 220 × 220 mm; matrix, 320 × 256; voxel size, 0.43 × 0.43 × 6 mm; interslice gap, 1.5 mm; number of slices 24; and number of averages 1.”

*#5 MRI preprocessing: “3D T1w images were linearly co-registered with 2D axial T2w images using SPM12. Brain masks were created by skull-stripping the co-registered T1w images using the BrainExtraction Tool with FSL (version 5.0.11) [20] and binarizing it with FSLmaths.”I was wondering why did the authors use SPM for coregistration only. It's better to use FSL or SPM, sometime the header information is not properly copied from one platform to other. However, it's possible to copy the headers from spm to fsl properly.*

Reply: We originally planned to run the same pipeline as described by Cooper G, et al. (Front Neurol 2019;10:334). However, when testing the pipeline with our available data, we found that FSL did not coregister 3D and 2D images together very accurately, whereas SPM12 was able to do this very successfully.

*#6 T1w/T2w calculation: The authors mentioned that for T1w/T2w quantification they used previously published technique by Misaki et al MRM 2014, but it is not clear how did they perform bias correction which is important for better quantification of T1w/T2w. Did they use their script or just follow their approach (it looks there is no publicly available tool online by Misaki et al)? Depending on the bias correction technique, T1w/T2w maps may contain residual radio frequency transmit field (B1+) biases, which may be correlated with these variables of interest, leading to potentially spurious results.*

Reply: We thank the reviewer for pointing out that bias correction is missing our pipeline. We are aware of the growing literature demonstrating the importance of B1+ correction for T1W/T2W at 3 T and 7 T, where B1+ is more inhomogeneous. However, this study used a 1.5-T scanner, where B1+ is more homogenous and the effect could be assumed to be smaller (see, for example, Figure 3 of Dieringer et al. 2014 PLOS ONE, <https://journals.plos.org/plosone/article?id=10.1371/journal.pone.0091318>.

We have added a statement indicating a limitation that bias correction is missing in our pipeline, as follows.

Page 21, line 355-359

“Finally, bias correction was missing in our pipeline of sT1W/T2W calculation. Bias correction is important for the sT1W/T2W ratio on high-magnetic-field MRI to avoid the effects of the inhomogeneity of the radiofrequency transmit (*B1^+^*) field. However, this study used a 1.5-T scanner, where *B1^+^* is more homogenous and the effect could be assumed to be smaller [49].”

In line with this revision, we have added a reference.

49. Dieringer MA, Deimling M, Santoro D, Wuerfel J, Madai VI, Sobesky J, et al. Rapid parametric mapping of the longitudinal relaxation time T1 using two-dimensional variable flip angle magnetic resonance imaging at 1.5 Tesla, 3 Tesla, and 7 Tesla. PLoS One. 2014;9:e91318.

*#7 To measure diagnostic accuracy by Youden ROC method, I suspect about the accuracy of the measurement with this small sample size.*

Reply: According to the reviewer’s comment, we have changed the method for determining the cutoff point for ROC analysis from the Youden method to the point on the ROC curve closest to the upper left corner. Moreover, we have added the small sample size as a limitation. With these changes, we have revised sentences in the Methods section and added sentences in the Results and Discussion sections, as follows.

Page 8, line 186-192

“All statistical analyses, except for receiver operating characteristic (ROC) curve analyses, were performed using Statistical Package for the Social Sciences (version 25.0; SPSS Inc., IBM Corp., Chicago, IL, USA). The ROC curve analyses and Smirnov–Grubb’s test were performed using EZR (Saitama Medical Center, Jichi Medical University, Saitama, Japan), which is a graphical user interface for R (The R Foundation for Statistical Computing, Vienna, Austria, version 2.13.0) [26]. More precisely, EZR is a modified version of R commander (version 1.6-3) designed to add statistical functions frequently used in biostatistics.”

Page 9, line 197-202

“For evaluating differences in MCP sT1W/T2W ratio values and MCP volume among the four groups, a one-way analysis of covariance was used using age as the covariate. The ability of the MCP sT1W/T2W ratio values and MCP volume to differentiate between groups of patients was assessed using ROC curve analyses, and the optimal cutoff point was determined as the point on the ROC curve closest to the upper left corner, as used by Holmes [27].”

Page 11, line 221-223

“The AUC for differentiating MSA-C from SCA-3 was 0.895 (95% confidence interval [CI], 0.794–0.995), with a sensitivity of 0.844 and specificity of 1.0.”

Page 11, line 224-227

“The AUC for differentiating MSA-C from controls was 0.960 (95% CI, 0.908–1.000), with a sensitivity of 0.906 and specificity of 0.941. The AUC for differentiating SCA-3 from SCA-6 was 0.898 (95% CI, 0.775–1.000), with a sensitivity of 0.875 and specificity of 0.812 (Fig. 3).”

Page 20, line 336

“This study has several limitations.”

Page 21, line 352-355

“Fourth, the sample size of the SCA3 group was small (n = 8), and future prospective studies with larger sample sizes that evaluate clinical scales for cerebellar ataxia are needed to validate the findings of this study and clarify the relationship between clinical severity of cerebellar ataxia and the MCP sT1W/T2W ratio.”

In line with these revisions, we have revised Figure 3 and added references.

26. Kanda Y. Investigation of the freely available easy-to-use software ‘EZR’ for medical statistics. Bone Marrow Transplant 2013;48:452-8.

27. Holmes W. A short, psychiatric, case-finding measure for HIV seropositive outpatients. Medical Care 1998;36:237-43.

*#8 Figure1: you haven’t mentioned what type of images (i.e., T2-weighted images) are in the figure. Please include the corresponding T1w/T2w maps with the signal intensity scale.*

Reply: According to the reviewer’s comments, we have revised Figure 1 and added a sentence in the Figure Legends section, as follows.

Page 7, line 171 to Page 8, line 172

“Upper row: axial T2-weighted images. Lower row: standardized T1-weighted/T2-weighted ratio maps”

*#9 Figure2: significant difference between MSA-C with SCA3 and SCA6 might be derived from the outliers of MSA-C data. I would use the outlier removal tool and compare the results.*

Reply: We thank the reviewer for the comment. We have added Smirnov–Grubb’s test for evaluating the outliers. Moreover, we have revised sentences in the Methods section and added a sentence in the Results section, as follows.

Page 8, line 188-191

“The ROC curve analyses and Smirnov–Grubb’s test were performed using EZR (Saitama Medical Center, Jichi Medical University, Saitama, Japan), which is a graphical user interface for R (The R Foundation for Statistical Computing, Vienna, Austria, version 2.13.0) [26].”

Page 9, line 212-213

“The Smirnov–Grubb’s test was used for evaluating the outliers of the MCP sT1W/T2W ratio value in each subject group.”

Page 11, line 227-231

“Even after excluding one patient with MSA-C with an outlier MCP sT1W/T2W ratio value, the MCP sT1W/T2W ratio values in patients with MSA-C were significantly lower than those in patients with SCA3 and SCA6 and controls (0.03 ± 0.06 vs. 0.13 ± 0.02, 0.17 ± 0.03, and 0.17 ± 0.03, respectively; *p* < 0.001).”

*#10 Discussion section should be more organized.*

Reply: According to the reviewer’s suggestion, we have revised sentences in the Discussion section, as follows.

Page 18, line 286-288

“We showed that the MCP sT1W/T2W ratio is highly sensitive and specific for not only distinguishing MSA-C from SCA3, SCA6, or controls but also differentiating SCA3 from SCA6.”

Page 19, line 309-310

“The usefulness of the MCP sT1W/T2W ratio in discriminating MSA-C from SCA3 in this study might be due to differences in the degree of MCP degeneration in the two diseases.”

Page 20, line 324-326

“The pathological background for this may be the difference in MCP degeneration, which is observed to some extent in SCA3, as described above, but is rarely observed in SCA6.”

*#11 It would be great to show the comparison of volumetric measure of the MCP indifferent disease conditions.*

Reply: We thank the reviewer for this important suggestion. The volume of the MCP was measured and compared between the groups. Moreover, we evaluated the correlation between MCP volume and MCP sT1w/T2w ratio value and compared the diagnostic accuracy between MCP volume and MCP sT1w/T2w ratio. Along with this revision, we have added sentences in the Methods and Results sections, as follows.

Page 8, line 174-183

“**MCP volume analysis**

The MCP volume was measured in all subjects by exploiting a validated probabilistic 3D atlas of the cerebellar white matter structure [24]. Parcellation at a 90% probability threshold was used. The atlas of the MCP was transformed from the Montreal Neurological Institute 152 space [20] to the individual subject’s space using the deformation parameters obtained from the normalization procedure using ANTs [23]. The mean volume value in the left and right MCPs was used as the MCP volume for each subject. For further analyses, the MCP volume was normalized by each subject’s intracranial volume. Values represent the ratio of the volume of the MCP divided by the subject-specific total intracranial volume (written as *m* × 10^-3^).”

Page 9, line 197-202

“For evaluating differences in MCP sT1W/T2W ratio values and MCP volume among the four groups, a one-way analysis of covariance was used using age as the covariate. The ability of the MCP sT1W/T2W ratio values and MCP volume to differentiate between groups of patients was assessed using ROC curve analyses, and the optimal cutoff point was determined as the point on the ROC curve closest to the upper left corner, as used by Holmes [27].”

Page 9, line 202-204

“The DeLong test was used to compare the area under curves (AUCs) between the MCP sT1W/T2W ratio and MCP volume to differentiate among the four groups.”

Page 9, line 210-212

“Pearson correlation analysis was performed to evaluate the relationship between the MCP sT1W/T2W ratio values and MCP volume in patients with MSA-C.”

Page 18, line 265-283

“The MCP volume, normalized by each subject’s intracranial volume, was significantly lower in patients with MSA-C patients than those in patients with SCA6 and controls (0.20 ± 0.06 vs. 0.31 ± 0.05 and 0.34 ± 0.05, respectively; *p* < 0.001). Moreover, the MCP volume was significantly lower in patients with SCA3 than those in patients with SCA6 and controls (0.23 ± 0.04 vs. 0.31 ± 0.05 and 0.34 ± 0.05, respectively; *p* < 0.001; *p* = 0.01 and *p* < 0.001).

The AUC value for differentiating MSA-C from SCA3 was 0.664 (95% CI, 0.486–0.842), with a sensitivity of 0.750 and specificity of 0.625. The AUC for differentiating MSA-C from SCA6 was 0.924 (95% CI, 0.852–0.995), with a sensitivity of 0.844 and specificity of 0.875. The AUC for differentiating MSA-C from controls was 0.967 (95% CI, 0.925–1.000), with a sensitivity of 0.906 and specificity of 0.941). The AUC for differentiating SCA3 from SCA6 was 0.914 (95% CI, 0.802–1.000), with a sensitivity of 0.875 and specificity of 0.875. The AUC of the MCP sT1W/T2W ratio was greater than that of the MCP volume for differentiating MSA-C from SCA3 (*p* = 0.0049). No significant differences were observed between the AUC of the MCP sT1W/T2W ratio and that of MCP volume for differentiating MSA-C from SCA6 and controls (*p* = 0.304 and 0.781, respectively). Moreover, no significant difference was observed between the AUC of the MCP sT1W/T2W ratio and that of MCP volume for differentiating SCA3 from SCA6 (*p* = 0.855). In patients with MSA-C, a significant correlation was observed between the MCP volume and MCP sT1W/T2W ratio (*r* = 0.732; *p* < 0.001).”

*#12 Please add the small sample size as a limitation.*

Reply: We agree with the reviewer’s concerns about the small sample sizes in our study. We have revised and added sentences in the Discussion section, as follows.

Page 20, line 336

“This study has several limitations.”

Page 21, line 352-355

“Fourth, the sample size of the SCA3 group was small (n = 8), and future prospective studies with larger sample sizes that evaluate clinical scales for cerebellar ataxia are needed to validate the findings of this study and clarify the relationship between clinical severity of cerebellar ataxia and the MCP sT1W/T2W ratio.”

*#13 “In conclusion, the MCP sT1w/T2w ratio is a highly sensitive imaging-based marker for detecting MSA-C related changes and might be useful for differentiating 274 MSA-C from SCA3 or SCA6.” With a relatively small sample size and poor quality of data (especially 2D T1w,T2w data, 1.5 mm vs. 6mm slice thickness collected at 1.5 T), it is tough to claim “highly sensitive imaging-based marker ….”*

Reply: We thank the reviewer for the comment. Accordingly, we have revised sentences in the Abstract and Conclusions sections, as follows.

Page 3, line 50-51

“The MCP sT1W/T2W ratio might be a sensitive imaging-based marker for detecting MSA-C-related changes and differentiating MSA-C from SCA3 or SCA6.”

Page 21, line 362 to Page 22, line 363

“In conclusion, the MCP sT1W/T2W ratio might be a sensitive imaging-based marker for detecting MSA-C-related changes and useful for differentiating MSA-C from SCA3 or SCA6.”
