# Supplementary material for: Diagnostic efficacy of the magnetic resonance T1w/T2w ratio for the middle cerebellar peduncle in multiple system atrophy and spinocerebellar ataxia: A preliminary study
Source: PLoS One. 2022 Apr 15;17(4):e0267024. doi: 10.1371/journal.pone.0267024 (PMC9012356; doi:10.1371/journal.pone.0267024)
Supplement: Attachment — Submitted filename: Response to Reviewers.docx [file pone.0267024.s003.docx]

# Reply to Reviewer 1

We greatly appreciate your helpful comments and suggestions. Changes made in accordance with reviewers’ comments are indicated in red font in the revised manuscript.

*#1 The authors have partly responded to my questions and made changes to the manuscript. I would thank the authors to add healthy controls. But the major concern is the data used and sample size. Therefore, it can be reported as preliminary findings and further investigation with a large sample size and standard T1 and T2 images is required to validate these findings. I have some comments:*

Reply: We thank the Reviewer for their important comment. Accordingly, we have revised the title.

Page1, line 1-3

“Diagnostic efficacy of the magnetic resonance T1w/T2w ratio for the middle cerebellar peduncle in multiple system atrophy and spinocerebellar ataxia: a preliminary study”

*#2 The authors followed the paper by Misaki et al 2015 but they didn’t perform bias correction. As I mentioned before that bias correction is an important step for T1w/T2w ratio mapping and depending on the bias correction technique, T1w/T2w maps may contain residual radiofrequency B1+ biases, which may be correlated with these variables of interest, leading to potentially spurious results. It's true that B1+ is more homogeneous at 1.5T compared to higher magnetic field strengths such as 3T or 7T (say center brightness artifact is ~30% at 3T while ~1.5% at 1.5T; Bernstein et al 2006), but not perfectly homogeneous and it’s still required to perform bias correction for T1w/T2w mapping even at 1.5 T MRI (see previous works at 1.5 T; Ganzetti et al 2016).*

Reply: We followed the reviewer's suggestion and added the bias collection step, which partially changed the results of the study. We strongly agree with the reviewer's comments on the importance of bias collection step and would like to express our deepest gratitude to the reviewer for suggesting the addition of bias collection. Accordingly, we have revised and added sentences in the Abstract, Methods, Results, and Discussion sections.

Page 2, line 41-48

“The MCP sT1w/T2w ratio showed high diagnostic accuracy for distinguishing MSA-C from SCA3 (area under curve = 0.934), SCA6 (area under curve = 0.965), and controls (area under curve = 0.980). The diagnostic accuracy of the MCP sT1w/T2w ratio for differentiating MSA-C from SCA3 or SCA6 (90.0% for MSA-C vs. SCA3, and 91.7% for MSA-C vs. SCA6) was comparable to or superior than that of visual interpretation of MCP hyperintensities (80.0–87.5% in MSA-C vs. SCA3 and 87.6–97.9% in MSA-C vs. SCA6) or the HCB sign (72.5–80.0% in MSA-C vs. SCA3 and 77.1–93.8% in MSA-C vs. SCA6).”

Page 6, line 123-125

“All T1w and T2w images were preprocessed as previously described before the sT1w/T2w ratio was calculated [16]; specifically, the intensity inhomogeneity correction was applied to the T1w and T2w images using *N4BiasFieldCorrection* [20].”

Page 12, line 226-238

“The MCP sT1w/T2w ratio values were significantly lower in patients with MSA-C than those in patients with SCA3 and SCA6 and controls (0.07 ± 0.06 vs. 0.17 ± 0.03, 0.18 ± 0.03, and 0.19 ± 0.03, respectively; *p* < 0.001) (Fig. 3). The AUC for differentiating MSA-C from SCA-3 was 0.934 (95% confidence interval [CI], 0.854–1.000), with a sensitivity of 0.906 and specificity of 0.875. The AUC value for differentiating between MSA-C and SCA-6 was 0.965 (95% CI, 0.922–1.000), with a sensitivity of 0.906 and a specificity of 0.938. The AUC for differentiating MSA-C from controls was 0.980 (95% CI, 0.951–1.000), with a sensitivity of 0.906 and specificity of 0.941. The AUC for differentiating SCA-3 from SCA-6 was 0.633 (95% CI, 0.388–0.878), with a sensitivity of 0.750 and specificity of 0.625 (Fig. 4). Even after excluding one patient with MSA-C with an outlier MCP sT1w/T2w ratio value, the MCP sT1W/T2W ratio values in patients with MSA-C were significantly lower than those in patients with SCA3 and SCA6 and controls (0.07 ± 0.05 vs. 0.17 ± 0.03, 0.18 ± 0.03, and 0.19 ± 0.03, respectively; *p* < 0.001).”

Page 13, line 263-265

“In patients with MSA-C, a significant inverse correlation between the extent of MCP hyperintensities and MCP sT1w/T2w ratio was observed (*r* = −0.832; *p* < 0.001).”

Page 16, line 267-274

“Diagnostic accuracy of the MCP sT1w/T2w ratio, visual interpretation of the MCP hyperintensities, and the HCB sign are listed in Table 3, and we found that the diagnostic performance of the MCP sT1w/T2w ratio for differentiating MSA-C from SCA3, SCA6, and controls was comparable to or better than that of visual interpretation of MCP hyperintensities and the HCB sign for all three reviewers. However, the diagnostic performance of visual interpretation of MCP hyperintensities and the HCB sign for differentiating SCA3 from SCA6 was comparable to or better than that of the MCP sT1w/T2w ratio.”

Page 19, line 286-292

“The AUC of the MCP sT1w/T2w ratio was greater than that of the MCP volume for differentiating MSA-C from SCA3 (*p* < 0.001). No significant differences were observed between the AUC of the MCP sT1w/T2w ratio and that of MCP volume for differentiating MSA-C from SCA6 and controls (*p* = 0.234 and 0.577, respectively). The AUC of the MCP volume was greater than that of the MCP sT1w/T2w ratio for differentiating SCA3 from SCA6 (*p* = 0.020).”

Page 19, line 292-293

“In patients with MSA-C, a significant correlation was observed between the MCP volume and MCP sT1W/T2W ratio (*r* = 0.619; *p* < 0.001).”

Page 19, line 296-297

“We showed that the MCP sT1W/T2W ratio is highly sensitive and specific for distinguishing MSA-C from SCA3, SCA6, or controls.”

Page 21, line 332-336

“The substantial discriminatory power of the MCP sT1w/T2w ratio and visual interpretation of MCP hyperintensities and the HCB sign described here implies that they may also be useful for differentiating between SCA3 and SCA6. The pathological background for this may be the difference in MCP degeneration, which is observed to some extent in SCA3, as described above, but is rarely observed in SCA6.”

Page 21, line 341-345

“The cohort of SCA3 and SCA6 patients included in this study is not large, and therefore, further studies that include data from a large cohort of SCA3 and SCA6 patients are needed to verify the usefulness of MCP sT1w/T2w ratio values and visual interpretation of MCP hyperintensities and the HCB sign in differentiating between SCA3 and SCA6.”

Page 22, line 362-365

“Finally, the sample size of the SCA3 group was small (n = 8), and future prospective studies with larger sample sizes that evaluate clinical scales for cerebellar ataxia are needed to validate the findings of this study and clarify the relationship between clinical severity of cerebellar ataxia and the MCP sT1W/T2W ratio.”

According to this revision, we have revised Figure 3 and Figure 4.

According to this revision, we have revised S1 Table.

According to this revision, we have added a reference.

20. Tustison NJ, Avants BB, Cook PA, Zheng Y, Egan A, Yushkevich PA, et al. N4ITK: improved N3 bias correction. IEEE Trans Med Imaging. 2010;29:1310-20.

*#3 It would be great to add a figure for a pipeline for creating a T1w/T2w image (i.e., summarize key steps with representative images in native and standard spaces).*

Reply: We thank the Reviewer for their important comment. Accordingly, we have added a pipeline for creating a sT1w/T2w ratio map as Figure 1.

Page 6, line 140-141

“Schematic representation of the pipeline for creating a sT1w/T2w ratio map is described in Figure 1.”

Page 7, line 152-154

“**Figure 1. Schematic representation of the pipeline for creating a sT1w/T2w ratio map.**

T1w, T1-weighted; T2w, T2-weighted; GM, gray matter: WM, white matter; sT2, a scaled T2-weighted image”

*#4 Figure 1: Why did the authors use images at different slice locations in Figure 1? I would recommend using the same slice locations for all cases in Figure 1. Also, show the corresponding images for T1w/T2w maps with the same image size.*

Reply: We thank the Reviewer for their important comment. Accordingly, we have revised Figure 2 and a sentence in Figure legend.

Page 8, line 173-175

“(A) Grade 0 (negative) HCB sign and absent MCP hyperintensities in a 74-year-old man with a SCA6 disease duration of 2.3 years.”

*#5 Abstract: “Methods” section was not updated for healthy controls.*

Reply: We thank the Reviewer for their careful observation and apologize for the oversight. The sentence has been revised as follows:

Page 2, line 34-35

“We included 32 MSA-C, 8 SCA type 3 (SCA3), 16 SCA type 6 (SCA6) patients, and 17 controls, and the MCP sT1w/T2w ratio was analyzed using a region-of-interest approach.”
